# Epidemic Keratoconjunctivitis-Associated Acute Dacryoadenitis in an Adult

**DOI:** 10.7759/cureus.27003

**Published:** 2022-07-19

**Authors:** Yasuhiro Takahashi, Aric Vaidya, Shinjiro Kono, Tatsuro Yokoyama, Hirohiko Kakizaki

**Affiliations:** 1 Oculoplastic, Orbital, and Lacrimal Surgery, Aichi Medical University Hospital, Aichi, JPN; 2 Oculoplastic, Orbital, and Lacrimal Surgery, Kirtipur Eye Hospital, Kathmandu, NPL

**Keywords:** corneal opacity, serologic evidence, steroid administration, epidemic keratoconjunctivitis, acute dacryoadenitis

## Abstract

A 39-year-old man presented with a five-day history of swelling of the right upper eyelid and ocular irritation in the right eye. On the first examination, the patient showed conjunctival injection, conjunctival chemosis, swollen upper eyelid, and palpable lacrimal gland with tenderness on the right side. Magnetic resonance images showed an inflamed right lacrimal gland. Blood test demonstrated negative results for immunoglobulin M of Epstein-Barr, mumps, herpes simplex, and herpes zoster viruses. We administered oral prednisolone (30 mg/day) based on a possible diagnosis of idiopathic dacryoadenitis. One week after steroid treatment, the periocular inflammation reduced to some extent although the inflammation substantially persisted. Four weeks after the steroid treatment, the patient informed us that he had met his friend 10 days before the onset, and that friend had conjunctival injection at that time which was subsequently diagnosed as an epidemic keratoconjunctivitis. The periocular inflammation subsided, but two corneal white spots were observed on slit-lamp examination. Although immunochromatographic test for adenovirus was negative, the blood test showed a positive result for immunoglobulin M of adenovirus serotype 3. In eight weeks of follow-up, the number of corneal opacities increased to five spots, but the acute dacryoadenitis did not recur.

## Introduction

Acute dacryoadenitis is an acute inflammation of the lacrimal gland caused by infectious, autoimmune, or idiopathic causes [1-3]. Although bacterial, autoimmune, and idiopathic acute dacryoadenitis is more common in adults, viral dacryoadenitis develops more frequently in children [3]. The most common viral etiology is the Epstein-Barr virus, and infections due to mumps, herpes simplex, and herpes zoster viruses are less common [1-3]. Viral dacryoadenitis is self-resolving, while bacterial infection requires antibiotic administration, and the autoimmune and idiopathic causes respond to steroids [1].

Adenovirus can cause both epidemic keratoconjunctivitis (EKC) and acute dacryoadenitis [2-4]. Most of the cases of acute dacryoadenitis caused by adenovirus involve the pediatric population [3,5,6], and the development of combined EKC and acute dacryoadenitis in adults is rare [7-11]. In this article, we report a case of acute dacryoadenitis associated with EKC in an adult.

## Case presentation

This study was conducted in accordance with the tenets of the Declaration of Helsinki and its later amendments. Written informed consent for publication of identifiable face photos was obtained from the patient.

A 39-year-old man presented with a history of eyelid swelling and ocular irritation on the right side for five days. One day after the onset, he consulted with an ophthalmologist at another clinic, and levofloxacin eyedrop was administered. However, the symptoms got worse the next day. The patient did not have clinical features of headache, fever, or fatigue.

**Figure 1:**
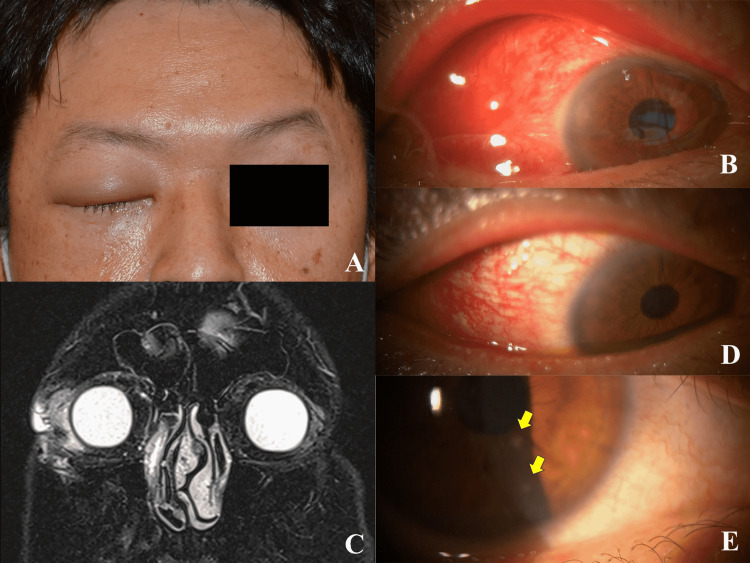
Case presentation (A) A face photo taken on the first examination showing a right upper eyelid swelling and edema. (B) Slit-lamp examination on the first examination revealing conjunctival injection and chemosis. (C) A T2-weighted fat-suppressed coronal magnetic resonance image showing a high-intensity lesion around an enlarged right lacrimal gland. (D) One week after oral steroid administration, conjunctival injection still persisted. (E) At eight weeks of follow-up, there are corneal opacities (arrows).

On the first examination, the best-corrected visual acuity was 1.0 in both eyes, and intraocular pressure was 17 mmHg in both eyes. The right upper eyelid was swollen (Figure 1, Panel A). The right lacrimal gland was palpable, and tenderness was present. The preauricular and cervical lymph nodes were not palpable. Slit-lamp examination revealed severe bulbar conjunctival injection and chemosis in the right eye (Figure 1, Panel B). The conjunctival injection was more severe in the temporal region. There was no fluorescein staining on the ocular surface. T2-weighted fat-suppressive magnetic resonance images showed a high-intensity area extending from an enlarged right lacrimal gland (Figure 1, Panel C). Blood tests demonstrated negative results for immunoglobulin M of Epstein-Barr, mumps, herpes simplex, and herpes zoster viruses. Rheumatoid factor, soluble interleukin-2 receptor, and immunoglobulin G4 were not elevated.

As we diagnosed acute dacryoadenitis possibly due to an idiopathic cause, we administered oral prednisolone (30 mg/day). One week after the steroid treatment, the conjunctival injection and eyelid swelling substantially persisted (Figure 1, Panel D). There was no corneal fluorescein staining. He still had tenderness on palpation of the right lacrimal gland. The dose of oral prednisolone was gradually tapered (5 mg reduction every two weeks).

Four weeks after the steroid treatment, the patient informed us that he had met his friend 10 days before the onset of symptoms, who had a conjunctival injection at that time, and it was later on diagnosed as EKC with immunochromatography. The conjunctival injection subsided, but there were two corneal white spots. The right lacrimal gland was not palpable, and there was no tenderness on palpation. The immunochromatographic test for adenovirus was negative. The blood test showed a positive result for immunoglobulin M of adenovirus serotype 3, although the blood tests of adenovirus serotypes 7, 8, 11, 19, and 37 were negative.

The patient sustained a right ring finger fracture while playing dodgeball with his son one week after the onset of acute dacryoadenitis. For treatment of the fracture, consultation with an orthopedic surgeon was done who stopped steroid administration that was continuing for the last five weeks. At eight weeks of follow-up, the number of corneal opacities increased to five spots (Figure 1, Panel E). Visual acuity was 1.0 in both eyes, and the acute dacryoadenitis did not recur.

## Discussion

We report a case of acute dacryoadenitis in an adult associated with EKC. There had been only four similar cases and one suspected case previously [7-11]. One reported patient aged 29 years showed a positive result of a lacrimal gland biopsy culture for adenovirus [7]. However, the herpes simplex virus seemed to be the more likely culprit of dacryoadenitis in this previous case based on the results of immunohistochemical studies [7,8]. Another reported patient aged 32 years showed a positive serologic test for adenovirus immunoglobulin M, a positive result of adenovirus polymerase chain reaction from a viral swab of the conjunctiva, and characteristic subepithelial corneal deposits, which were strongly supportive pieces of evidence of the association between acute dacryoadenitis and EKC [8]. A magnetic resonance imaging study showed a patient aged 36 years with acute conjunctivitis, dacryoadenitis, and dacryocystitis [9]. Adenovirus serotype 8 was isolated from this patient [9]. A patient aged 36 years was only clinically diagnosed with EKC and acute dacryoadenitis without serologic evidence or virus isolation [10]. In one suspected 67-year-old patient with EKC and acute orbital inflammation, adenovirus infection was diagnosed before referral to the authors [11].

Our patient in this case who is 39 years old showed a positive serologic result for immunoglobulin M of adenovirus serotype 3. Immunochromatography for adenovirus was negative, but these tests were performed four weeks after the first examination, and dacryoadenitis and conjunctival injection had already subsided at that time. The patient met his friend with EKC 10 days before the onset of symptoms. As the incubation period of EKC ranges from five to 14 days, the period from the encounter to the onset of symptoms coincided with the incubation period of EKC. If the cause of this dacryoadenitis was idiopathic or due to autoimmune status, the inflammation would have subsided shortly after starting the oral prednisolone. Late-onset corneal opacities also supported the diagnosis of EKC-associated acute dacryoadenitis in this case.

Possible mechanisms of development of EKC-associated acute dacryoadenitis are a direct invasion of adenovirus into the lacrimal gland and secondary spread of adenovirus from keratoconjunctival lesions [6,9]. In this case, adenovirus likely invaded the conjunctival sac, similar to the cases with EKC, and the virus secondarily spread to the lacrimal gland. Considering the direction of tear flow, secondary adenoviral infection of the lacrimal drainage system is more common. Although acute dacryocystitis is rare and there had been only three reported cases [9,12], adenovirus is actually a common etiology of obstruction of the lacrimal drainage system [13].

We administered oral prednisolone because of a possible diagnosis of idiopathic acute dacryoadenitis. In previous reports with similar cases, an oral steroid was administered for reducing the lacrimal gland inflammation [8,10,11]. However, viral dacryoadenitis is a self-limiting disease [1]. In addition, there is no effective treatment for EKC, and steroid administration may therefore not be necessary.

## Conclusions

In conclusion, we report a case of EKC-associated acute dacryoadenitis in an adult. A positive serologic result and clinical evidence supported its diagnosis. There had been four similar cases and one suspected case previously, although two of them did not show serologic evidence or confirmation of virus isolation. As viral dacryoadenitis has a self-limiting nature, the necessity of steroid administration is a debatable issue for this case.
